# Kainic Acid-Induced Excitotoxicity Experimental Model: Protective Merits of Natural Products and Plant Extracts

**DOI:** 10.1155/2015/972623

**Published:** 2015-12-17

**Authors:** Nur Shafika Mohd Sairazi, K. N. S. Sirajudeen, Mohd Asnizam Asari, Mustapha Muzaimi, Swamy Mummedy, Siti Amrah Sulaiman

**Affiliations:** ^1^Department of Chemical Pathology, School of Medical Sciences, Universiti Sains Malaysia, Health Campus, Kubang Kerian, 16150 Kota Bharu, Kelantan, Malaysia; ^2^Department of Anatomy, School of Medical Sciences, Universiti Sains Malaysia, Health Campus, Kubang Kerian, 16150 Kota Bharu, Kelantan, Malaysia; ^3^Department of Neurosciences, School of Medical Sciences, Universiti Sains Malaysia, Health Campus, Kubang Kerian, 16150 Kota Bharu, Kelantan, Malaysia; ^4^Department of Pharmacology, School of Medical Sciences, Universiti Sains Malaysia, Health Campus, Kubang Kerian, 16150 Kota Bharu, Kelantan, Malaysia

## Abstract

Excitotoxicity is well recognized as a major pathological process of neuronal death in neurodegenerative diseases involving the central nervous system (CNS). In the animal models of neurodegeneration, excitotoxicity is commonly induced experimentally by chemical convulsants, particularly kainic acid (KA). KA-induced excitotoxicity in rodent models has been shown to result in seizures, behavioral changes, oxidative stress, glial activation, inflammatory mediator production, endoplasmic reticulum stress, mitochondrial dysfunction, and selective neurodegeneration in the brain upon KA administration. Recently, there is an emerging trend to search for natural sources to combat against excitotoxicity-associated neurodegenerative diseases. Natural products and plant extracts had attracted a considerable amount of attention because of their reported beneficial effects on the CNS, particularly their neuroprotective effect against excitotoxicity. They provide significant reduction and/or protection against the development and progression of acute and chronic neurodegeneration. This indicates that natural products and plants extracts may be useful in protecting against excitotoxicity-associated neurodegeneration. Thus, targeting of multiple pathways simultaneously may be the strategy to maximize the neuroprotection effect. This review summarizes the mechanisms involved in KA-induced excitotoxicity and attempts to collate the various researches related to the protective effect of natural products and plant extracts in the KA model of neurodegeneration.

## 1. Introduction

Neurodegeneration involves the progressive loss of structure and function of neurons. Various types of biological mechanism have been implicated in neurodegeneration. Excitotoxicity is considered to be a major mechanism of neuronal death in acute and chronic neurodegenerative diseases, such as Alzheimer's disease (AD), Parkinson's disease (PD), Huntington's disease (HD), temporal lobe epilepsy (TLE), and amyotrophic lateral sclerosis (ALS) [1]. The concept of “excitotoxicity” was formulated by Olney in 1969 [2] which was referred to as a neuronal degeneration triggered by the over- or prolonged activation of glutamate receptors in the central nervous system (CNS) by excitatory amino acids.

Glutamate is a major excitatory neurotransmitter that mediates fast synaptic transmission and plays an important role in the mammalian CNS (brain and spinal cord) [3, 4]. Excess glutamate is highly toxic to neurons. Glutamate acts through glutamate receptors. There are two major classes of glutamate receptors: ionotropic glutamate receptors (iGLURs) and metabotropic glutamate receptors (mGLURs). These glutamate receptors differed in terms of their functionality. iGLURs mediate fast postsynaptic potentials by activating ion channels directly, while mGLURs mediate slow postsynaptic potentials by coupling to intracellular G proteins and second messengers [5, 6]. iGLURs can be divided into three subtypes: N-methyl-D-aspartic-acid (NMDA) receptors, *α*-amino-3-hydroxy-5-methyl-4-isoxazole propionate (AMPA) receptors, and kainate receptors. They are generally named after their specific ligand and by the types of their activating agonists [7, 8].

In the animal models of neurodegeneration, excitotoxicity is commonly induced experimentally by chemical convulsants, particularly by kainic acid (KA) [9]. Administration of KA has widely been used as a tool to explore the mechanism involved in excitotoxicity.

## 2. Kainic Acid-Induced Excitotoxicity Model

**Figure 1:**
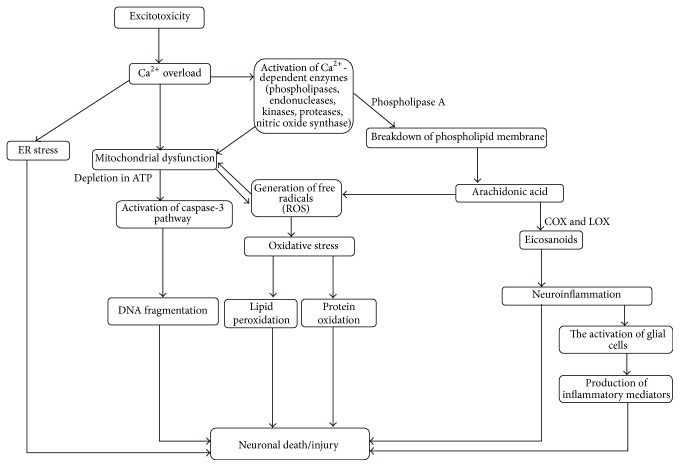
Proposed mechanism of action in KA-induced excitotoxicity.

KA [2-carboxy-4-(1-methylethenyl)-3-pirrolidiacetic acid] is a structural L-analog of glutamate and an agonist of kainate subtype of ionotropic glutamate receptors. KA exerts its neuroexcitatory property by binding to kainate receptors, which have presynaptic modulatory and postsynaptic excitatory actions [10, 11]. KA activates glutamate receptors and the overactivation of glutamate receptors produces neuronal membrane depolarization. This causes the influx of calcium ion (Ca^2+^) and subsequently triggers excitotoxic neuronal death cascade events (refer to Figure 1).

Studies in KA-induced animal experimental model have shown that administration of KA resulted in seizures [12–15], behavioral changes of rodents [16–22], oxidative stress [23–25], glial activation [26–32], production of inflammatory mediators [32, 33], endoplasmic reticulum (ER) stress [34–37], mitochondrial dysfunction, and selective neuronal degeneration in the brain of rodents [15, 36, 38–40].

Administration of KA is known to induce a sequence of well-characterized seizure syndromes and has resulted in behavioral changes of rodents including motor and cognitive performance [15, 17–20]. A single systematic injection of a convulsive dose of KA has resulted in limbic status epilepticus (SE), initiating neuropathological changes in limbic brain areas and subsequently long-term spontaneous recurrent seizures (SRSs) in both rat [40] and mice [41] as well as neuropathological lesions reminiscent of those found in patients with TLE [15, 42]. KA-induced SE causes irreversible neuronal degeneration in the selective brain areas, particularly in limbic structures (i.e., in the CA1 and CA3 regions of hippocampus and the hilus of dentate gyrus (DG)) [15].

Several studies have also demonstrated that there are behavioral changes in rodents after KA administration that resulted in memory deteriorations in the elevated plus-maze [16], increase in the activity in the open field test [21], and cognitive impairment in the passive avoidance test [22] and in Morris water maze task [17–20].

Oxidative stress may have a contributory role in neuronal and glial cell death [23–25, 43, 44]. There are growing evidences to suggest that oxidative stress has been implicated in the mechanism of excitotoxicity on different brain regions after the induction of KA on rodents [23–25]. The brain is considered to be very vulnerable to oxidative stress because of its great consumption of energy, oxygen, and glucose, large amount of peroxidizable polyunsaturated fatty acids, and relatively low antioxidant capability [45].

Oxidative stress occurs when there is disturbance in balance between antioxidant mechanism and the production of free radicals and redox status. KA acts on and activates kainate receptors to incite the influx of intracellular calcium. The entry of intracellular calcium can stimulate the formation of free radicals.

Overactivation of glutamate receptors by KA has resulted in the increased production of reactive oxygen species (ROS), the mediators of oxidative stress [24]. Oxidative stress can cause cellular damage and generation of ROS, which oxidizes membrane lipids, protein, and DNA. Increased level of intracellular Ca^2+^ also leads to the activation of several Ca^2+^-dependent enzymes [46]. Those enzymes include proteases (responsible for breaking down membrane and cytoskeletal proteins), endonucleases (responsible for DNA fragmentation), kinases, phospholipases (responsible for membrane damage), phosphatases, and nitric oxide synthase (NOS) [46–48].

Glial activation and neuroinflammation are believed to contribute to the development and progression of acute and chronic neurodegeneration [49–53]. Upon neuronal injury, neurons interact with glial cells (i.e., astrocytes and microglia). The survival of neurons and the postinjury repair of neurons are influenced by the activity of astrocytes and microglia. The activation of glial cells (as measured by increased activation of microglia and astrocytes) is associated with neuronal death upon KA administration [30–32]. Systemic injection of KA on rats has resulted in large increase of reactive astrocytes and microglial cell [54]. Activated microglia and astrocytes produced a large amount of inflammatory mediators, such as nitric oxide (NO), interleukin-1 beta (IL-1*β*), and tumor necrosis factor-alpha (TNF-*α*) [55], which influence the outcome of neurodegeneration [32, 33].

The overactivation of glutamate receptors by KA can also cause the fragmentation of ER membrane and ER stress with the activation of ER proteins like binding immunoglobulin protein (BiP, also known as glucose-regulated protein 78/GRP78), CCAAT/enhancer-binding protein- (C/EBP-) homologous protein (CHOP, also known as growth arrest and DNA damage inducible gene 153/GADD153), and caspase-12, which are involved in the neuronal apoptosis [34, 35].

Excessive influx of Ca^2+^ into neurons through ionic channels and generation of free radicals also cause the accumulation of Ca^2+^ and mitochondrial dysfunction, which leads to the collapse of potential at the mitochondrial inner membrane. This results in the mitochondrial swelling and the release of mitochondrial factors at the mitochondrial inner membrane space. The release of mitochondrial factors also triggers the activation of caspase and proteases that are responsible for the activation of apoptotic neuronal death. This leads to the cleavage of essential cellular substrates such as poly(ADP-ribose) polymerase-1 (PARP-1).

In a study by Gilliams-Francis et al. the intracerebral injection of KA has resulted in DNA damage, PARP-1 activation, and neuronal death [56]. The work suggested that there is a link between activation of caspase pathways and excitotoxic cell death and the neurons undergo caspase-mediated death, involving the DNA fragmentation and cleavage of PARP-1.

KA administration also causes mitochondrial dysfunction. Excessive generation of ROS causes reduction in energy level (depletion in ATP) and lipid peroxidation which leads to mitochondrial dysfunction [36]. These alterations in the mitochondrial function could be an early event prior to neuronal cell death.

## 3. The Mechanism of Preventive and Therapeutic Treatment Approaches in Neurodegeneration

Since excitotoxicity is an important process in the pathogenesis of neurodegeneration, neuroprotection seems promising for the preventive and therapeutic approaches in neurodegenerative diseases. Neuroprotection offers the potential to ameliorate or delay the process of neurodegeneration or to slow the rate of neurodegeneration through the interaction with the pathological changes process as well as the progression of clinical manifestations of the neurodegeneration diseases.

Considering the implication of oxidative stress in the mechanism of excitotoxicity-associated neurodegeneration, antioxidants and anti-inflammatory agents serve as potential candidates for neurodegeneration preventive and therapeutic treatment. Antioxidants would serve as agents that can inhibit the production of free radicals, interfere with formed free radicals, and limit the degree of damage to neurons [57].

In addition, inflammation can enhance the neuronal death and neuronal degeneration through the production of inflammatory mediators, such as cytokines and prostaglandin. The reduction of inflammation via cyclooxygenase-2 (COX-2) and 5-lipoxygenase (5-LOX) activities could also decrease inflammatory molecules, including prostanoids. Glia-derived cytokines can also counteract inflammation to block the unique signal transduction of specific proinflammatory cytokines and can also modify the outcome of neurodegeneration progression.

**Figure 2:**
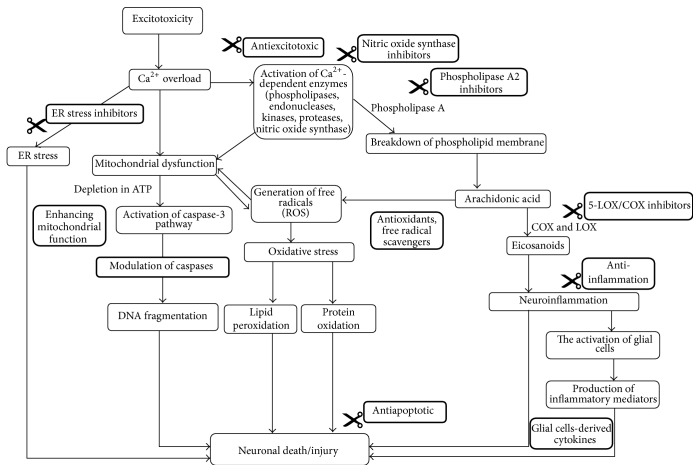
Illustrations of proposed mechanism of preventive and therapeutic treatment approaches in neurodegeneration.

Other potential approaches for treatment of neurodegenerative diseases are to improve the function of mitochondria and ER to inhibit the ER stress and apoptosis. A short summary and illustration of the proposed mechanism of action for the preventive and therapeutic strategies for neurodegenerative diseases are presented in Table 1 and Figure 2. Combination of multiple agents that target multiple pathways may result in synergistic effects to bring additive neuroprotective effect.

## 4. The Protective Effect of Natural Products and Plant Extracts in the KA Model of Neurodegeneration

For decades, many efforts attempted to elucidate the mechanism of excitotoxicity and neurodegeneration and to investigate its pharmacological interventions. Recently, there has been an emerging trend to search for natural resources to combat against neurodegenerative diseases. Reports on the potential beneficial effects of natural products and plant extracts in the experimental treatment of neurodegeneration continue to expand, largely on the effect by various constituents, including polyphenols for a wide range of medicinal, pharmacological, and biological properties. The following are summaries of the various reported studies on selected natural products and plant extracts involving the KA-induced experimental neurodegeneration model, namely, ginseng,* Uncaria rhynchophylla*, tea, and honey bee propolis.

### 4.1. Ginseng (*Panax sp.*)

Ginseng is the dried root of several species from* Panax *genus (Araliaceae family). There are seven major species to* Panax* genus but* Panax ginseng* (Asian ginseng),* Panax quinquefolius *(American ginseng), and* Panax japonicus *(Japanese ginseng) are the three most widely studied species [58–60]. The major active components found in ginseng are ginsenosides (steroidal saponins) [61]. The rest are polysaccharides, peptides, polyacetylenic alcohol, and fatty acids [58, 60, 61]. Ginsenosides have been isolated and classified into three groups, based on chemical structure of their sapogenins (aglycones): the panaxadiols group (i.e., Rb_1_, Rb_2_, Rb_3_, Rc, Rd, Rg_3_, Rh_2_, and Rh_3_), the panaxatriols group (i.e., Re, Rf, Rg_1_, Rg_2_, and Rh_1_), and the oleanolic acid group (i.e., Ro) [62]. Ginseng has been shown to possess antioxidants and anti-inflammatory properties [58]. Pharmacological effects of ginseng have been demonstrated on the CNS, with stimulatory effects and neurotransmission modulation [63].

In KA-induced excitotoxicity model, Lee et al. work in 2002 was the first to suggest that ginseng may have anticonvulsant activity [64]. It reported that KA-induced seizure in animal pretreated with a mixture of ginsenosides had shorter duration than in KA-only treated animals. This was supported by Shin et al. work [65], where repeated treatment with ginsenosides mixture before administration of KA has significantly reduced the number of wet dog shakes (WDS), delayed the onset of seizures, and decreased the score of seizures [65]. In another study by Lian et al., it has been demonstrated that the onset of KA-induced seizures was delayed and the score of seizures was decreased in animals pretreated with the partial purified Rb ginsenosides (Rb extract), significantly [60]. These results indicated that ginseng particularly with the presence of Rb ginsenosides suppresses KA-induced seizures and has significant anticonvulsant property.

Moreover, pretreatment with Rb extract before KA administration has reduced the percentage of animal having immunoreactivity for heat-shock protein-72 (HSP-72) [60]. Pretreatment with ginsenosides mixture before KA administration has also suppressed the induction of HSP-70 and has attenuated the neuronal cell death in the CA1 and CA3 regions of the hippocampus [64].

In addition, the treatment with the mixture of ginsenosides has significantly reduced the rise in KA-induced protein oxidation and lipid peroxidation and has significantly attenuated KA-induced glutathione oxidation in the homogenates and mitochondrial fraction of the hippocampus. The effect was more apparent in the mitochondrial fraction than in the homogenate of the hippocampus [65]. Mixture of ginsenosides also attenuated the decrease in manganese-superoxide dismutase-like immunoreactivity (SOD-2-IR) and in superoxide dismutase-2 (SOD-2) protein level in the CA1 and CA3 regions of the hippocampus [65] since Mn-SOD could protect mitochondria from superoxide radicals and the damage induced by KA-induced oxidative stress. This indicated that ginsenosides could prevent KA-induced excitotoxicity by attenuating oxidative stress, particularly in mitochondria, through its antioxidant mechanism.

Mixture of ginsenosides had also significantly attenuated the increase in intramitochondrial Ca^2+^ level and the decrease in mitochondrial transmembrane potentials in the hippocampus [65]. These findings implied that the mixture of ginsenosides of ginseng could reduce or protect against the excitotoxic effect of KA by attenuating the mitochondrial dysfunction.

Upon KA administration, a significant astrocyte and microglial response was observed and Rb fraction significantly inhibits the activation of microglia against KA-induced excitotoxicity [26]. Rb fraction also has been shown to prevent the hippocampal-dependent impairment of spatial cognitive function and hippocampal neurodegeneration [26]. This indicated that Rb fraction could protect neuron and glial cells against excitotoxicity induced by KA.

In a study on red ginseng extract (RGE), it has been shown that RGE decreased the production of ROS in KA-exposed primary hippocampal neuronal cell culture and inhibited the lipid peroxidation in hippocampal tissue [66]. This indicated that RGE can protect neurons from excitotoxicity through its antioxidant mechanism. Moreover, RGE has also been shown to attenuate the elevation of intracellular Ca^2+^ level and inhibit neuronal cell loss in KA-induced excitotoxicity* in vitro *model [66]. Excess accumulation of intracellular Ca^2+^ can initiate the excitotoxic process, leading to neuronal damage or death. By decreasing the elevation of intracellular Ca^2+^ level, RGE can protect neurons from neuronal damage or death.

These results suggested that ginseng, particularly with the presence of ginsenosides, displays neuroprotective and antioxidant effects against KA-induced excitotoxicity.

### 4.2. Uncaria

*Uncaria rhynchophylla* (Miq.) Jacks (UR) is the dried stems of* Uncaria*, a genus plant species from Rubiaceae family. UR is a medicinal herb used in the traditional Chinese medicine (TCM) to treat neuronal-associated diseases. Active components found in the extract of UR are the alkaloids of UR, which are rhynchophylline (RP), isorhynchophylline, hirsutine, hirsuteine, corynantheine, corynoxine, and dihydrocorynantheine [67, 68]. Among these alkaloids, RP and isorhynchophylline are the most widely studied and have been known as neuroprotective compounds [69].

The extract of UR has been shown to possess anticonvulsive effect and free radical scavenging activity in KA-induced epileptic seizures with the inhibition of lipid peroxidation [70, 71]. In addition, UR extract has reduced the spread of mossy fibers sprouting, an indicator of recurrent epilepsy [72]. Pretreatment with UR extract before KA administration also has increased the survival of neurons and reduced the epileptiform discharges in the hippocampus [29].

UR also has been reported to exhibit neuroprotective effect against KA-induced neuronal damage, associated with the reduction of microglial activation, neuronal nitric oxide synthase (nNOS), inducible nitric oxide synthase (iNOS), and apoptosis [73] and the attenuation of glial fibrillary acidic protein (GFAP) and S100 calcium-binding protein B (S100B) expression in the hippocampal region [29, 72]. This suggested that UR can prevent hippocampal neuronal death.

Collectively to date, these findings suggest that UR and RP display neuroprotective and anticonvulsive action in protecting neuronal damage and suppressing KA-induced seizures through multiple signaling pathways and therapeutic targets.

### 4.3. Tea

Tea is made from leaves and stem of* Camellia sinensis* plant. This plant is the same plant that is used for making nonfermented (fresh green), semifermented (oolong), fermented (black), and postfermented (Pu-Erh) tea. The chemical composition of tea contains many polyphenolic compounds, called green tea polyphenols. This includes catechins, theaflavins, tannins, and flavonoids. The most major green tea polyphenols are catechins, which include (−)-catechin (EC), (−)-epicatechin gallate (ECG), (−)-epigallocatechin (EGC), and (−)-epigallocatechin gallate (EGCG). Among those catechins, EGCG is the most active polyphenol. EGCG is higher in green tea and is responsible for the green tea effect [74].

In KA-induced seizures, fresh green tea leaf [74] and Pu-Erh tea leaves [75] extracts have attenuated the maximal seizure classes, the behavioral seizure patterns, and lipid peroxidation. While* in vitro*, these tea leaf extracts have reduced Ca^2+^ release, ROS production, and lipid peroxidation. These observations implied that fresh green tea leaves and Pu-Erh tea leaves extract attenuated oxidative stress and have anticonvulsive effect.

In the same studies, fresh green tea leaf and Pu-Erh tea leaf extracts appeared to reduce COX-2 and p38 mitogen-activated protein kinases (MAPK) expression and have reduced PGE2 production KA-induced* in vitro *PC12 cells [74, 75]. Thus, the tea leaf extract has potential neuroprotective and anticonvulsive effects against excitotoxicity.

### 4.4. Honey Bee Propolis

Honey bee propolis is a resinous mixture that honey bees collect from a variety of botanical sources. It has been used as a sealant for beehive. The chemical composition of propolis varies with geographic origin depending on the specificity of the local flora, the phenology of the source of plants, and the characteristics of climate [76].

Propolis has been shown to have a wide range of biological activities, including anti-inflammatory [77] and antioxidant [78–80], that are attributed chiefly by the presence of flavonoids [79, 81] and caffeic acid phenyl ester (CAPE) [80]. There are studies to suggest the role of flavonoids and CAPE in the antioxidant and anti-inflammatory activities of propolis [79–83].

Propolis has long been used as a folk medicine and protective remedy [84, 85]. In KA-induced excitotoxicity model, pretreatment with ethanol-extracted propolis before KA administration has reduced the increase of NO production along with the increase of thiobarbituric acid reactive substances (TBARS) production and the decrease of total antioxidant status (TAS) level [86], indicating that propolis supplementation ameliorated KA-induced oxidative stress.

Furthermore, propolis has been reported to attenuate proinflammatory cytokine marker, the TNF-*α* level following the administration of KA [84], suggesting that propolis can protect against KA-induced neuronal damage. Propolis also has been shown to restore glutamine synthase activity [86] and ameliorate caspase-3 and NOS activities [84] in the cerebellum, cerebral cortex, and brain stem regions of KA-induced animals. These findings demonstrated that propolis supplementation has beneficial effect against KA-induced neurodegeneration due to its antioxidant, anti-inflammatory, and antiapoptotic properties.

Moreover, propolis has been shown to protect against convulsive behavior induced by KA in a dose-dependent manner [85]. This suggests that propolis may also possess anticonvulsants property. The pretreatment with propolis also significantly prevented KA-induced neuronal loss in the CA1 and CA3 regions of the hippocampus [85].

### 4.5. Other Natural Products and Plant Extracts

Many other studies have also tested or reported on the protective effect of KA-induced excitotoxicity* in vivo* and* in vitro* models involving other natural products and plant extracts (as summarized in Table 2). Thus, natural products and plant extracts could be potential candidates in the preventive and efficient treatment of excitotoxicity-associated neurodegeneration diseases.

Collectively, these findings suggest that the natural products and plant extracts appear to have potential neuroprotective effect against KA-induced excitotoxicity through various mechanisms, primarily through their antioxidant, anti-inflammatory, and anticonvulsive activities. This signifies the therapeutic merits of the natural products and plant extracts as neuroprotective agents. Further studies are needed to determine the other potentials and various mechanisms of actions of these natural products and plant extracts guard against KA-induced excitotoxicity.

## 5. Conclusion

Natural products and plant extracts appear to offer potential beneficial effects on the CNS, particularly their neuroprotective effect against excitotoxicity. In addition, natural products and plant extracts provide promising avenue for further research to guard against development and progression of acute and chronic neurodegeneration. Further work can aim at targeting simultaneous pathways that underlie the various mechanisms involved in order to expand the therapeutic yields for various neurodegeneration diseases.

## Figures and Tables

**Table 1:** Proposed mechanisms of preventive and therapeutic treatment approaches in neurodegeneration.

Proposed mechanisms	Reference(s)
Anti-inflammation	[87–95]
Antioxidant activity	[25, 96–102]
Anticonvulsion and antiepileptic	[14, 15, 42, 64, 70, 72, 103, 104]
Modulation of apoptosis-related genes/proteins and signaling pathways	[74, 75, 84, 104–111]
Cognitive enhancer	[17–20, 22]
Manipulation of glial activation and inflammatory cytokines	[29, 72, 112–115]
Combating excitotoxicity	[116]
Enhancing mitochondrial functions	[36, 111]
Inhibition of ER stress by small molecular compounds	[34–37]
Inhibition of Phospholipase A by Phospholipase A inhibitors	[117]
Inhibition of NO by NOS inhibitors	[118]

**Table 2:** A summary on the protective effect of natural products and plant extracts against KA-induced excitotoxicity *in vivo and in vitro *experimental models.

Proposed mechanisms	Natural products/extracts/compounds	Sources	Experimental model	Reference(s)
Antioxidant activity	Decursin (purified from ethanol extract of the *Angelica gigas * Nakai root)	*Angelica gigas * Nakai	KA-induced *in vivo* and *in vitro* primary hippocampal neuronal cells excitotoxicity	[108]
	*Asparagus racemosus *extract	*Asparagus racemosus*	KA-induced *in vivo* excitotoxicity	[119]
	Butanol fraction of methanol extract from *Aster scaber* Thunb. leaves	*Aster scaber* Thunb. (Korean chamchwi)	KA-induced *in vivo* excitotoxicity	[96, 120]
	Acetyl-11-keto-*β*-boswellic acid (AKBA)	*Boswellia serrata*	KA-induced *in vivo* excitotoxicity	[95]
	Green tea leaf extract and gallic acid	*Camellia sinensis*	KA-induced *in vivo* excitotoxicity and KA-induced *in vitro *PC12 cells excitotoxicity	[74]
	Pu-Erh tea leaf extract and gamma-aminobutyric acid (GABA)	*Camellia sinensis *var. *assamica*	KA-induced *in vivo* excitotoxicity and KA-induced *in vitro *PC12 cells excitotoxicity	[75]
	Capsaicin	*Capsicum* (hot pepper)	KA-induced *in vivo* excitotoxicity	[121]
	Apigenin (flavone)	*Cirsium japonicum *and* Carduus crispus*	KA-induced *in vivo* and *in vitro* primary hippocampal neuronal cells excitotoxicity	[122]
	Curcumin	*Curcuma longa* Linn (turmeric)	KA-induced *in vivo* excitotoxicity	[123]
	Ursolic acid	*Cynomorium songaricum *Rupr. (Souyang)	KA-induced *in vitro *hippocampal neuronexcitotoxicity	[124]
	Galantamine	*Galanthus nivalis*	KA-induced *in vivo* excitotoxicity	[20]
	*Gastrodia elata* extract	*Gastrodia elata*	KA-induced *in vivo* excitotoxicity	[70, 125]
	Anthocyanins	*Glycine max *(L.) Merr. seed (Korean black bean)	KA-induced *in vitro * HT22 cells and primary hippocampal neuronal cells excitotoxicity	[111]
	Licorice flavonoids extracted→liquiritigenin, isoliquiritigenin, and liquiritin	*Glycyrrhiza uralensis*	KA-induced *in vivo* excitotoxicity	[18]
	Naringin (flavonoid)	Grapefruit and citrus fruit	KA-induced *in vivo* excitotoxicity	[22]
	Vineatrol	Grapes	KA-induced *in vivo* excitotoxicity	[126]
	Water fraction from methanol extract of *Ixeris dentata*	*Ixeris dentata*	KA-induced *in vivo* excitotoxicity	[127]
	Ginsenosides	*Panax ginseng *(Asian)	KA-induced *in vivo* excitotoxicity	[65]
	Red ginseng extract	*Panax ginseng *(Asian)	KA-induced *in vivo* and *in vitro *primary hippocampal neuron cell excitotoxicity	[66]
	Butanol fraction of methanol extract from *Petasite japonicus *Max. leaves (BMP) and its subfractions (BMP-I or BMP-II)	*Petasite japonicus *(Sieb. et Zucc.) Maxim. (Compositae) leaves	KA-induced *in vivo* excitotoxicity	[128]
	Petaslignolide A (lignin glycoside) butanol fraction of methanol extract from *Petasite japonicus* (Sieb. et Zucc.) Maxim. (Compositae) leaves	*Petasite japonicus* (Sieb. et Zucc.) Maxim. (Compositae) leaves	KA-induced *in vivo* excitotoxicity	[129]
	Ethanolic-extracted propolis	Honey bee propolis	KA-induced *in vivo* excitotoxicity	[84, 85]
	trans-Resveratrol	Red Grapes	KA-induced *in vivo* excitotoxicity	[130, 131]
	Sesamin	*Sesamum indicum* (sesame seeds)	KA-induced *in vivo* excitotoxicity and KA-induced *in vitro *PC12 cells and BV2 cells excitotoxicity	[132]
	Rhynchophylline and *Uncaria rhynchophylla *extract	*Uncaria rhynchophylla*	KA-induced *in vivo* and *in vitro *excitotoxicity	[70]
	Branch and leaf ethanol extracts of *Vitis thunbergii* var. *taiwaniana*	*Vitis thunbergii *var. *taiwaniana*	KA-induced *in vivo* and *in vitro *BV2 cells excitotoxicity	[133]
	*Withania somnifera* extract	*Withania somnifera*	KA-induced *in vivo* and *in vitro *excitotoxicity	[134]
	(−)-Epigallocatechin-3-gallate (EGCG)	Tea	KA-induced *in vivo* excitotoxicity	[135]

Anti-inflammation	*Green tea leaf extract and gallic acid*	*Camellia sinensis*	*KA-induced in vivo excitotoxicity and KA-induced in vitro PC12 cells excitotoxicity*	[74]
	*Pu-Erh tea leaf extract and gamma-aminobutyric acid (GABA)*	*Camellia sinensis *var.* assamica*	*KA-induced in vivo excitotoxicity and KA-induced in vitro PC12 cells excitotoxicity*	[75]
	Capsaicin	*Capsicum* (hot pepper)	KA-induced *in vivo* excitotoxicity	[121]
	Curcumin	*Curcuma longa* Linn (turmeric)	KA-induced *in vivo* excitotoxicity	[110]
	Sesamin	*Sesamum indicum* (sesame seeds)	KA-induced *in vivo* excitotoxicity and KA-induced *in vitro *PC12 cells and BV2 cells excitotoxicity	[132]
	Galantamine	*Galanthus nivalis*	KA-induced *in vivo* excitotoxicity	[20]
	Glycyrrhizin (triterpene)	*Glycyrrhiza glabra (licorice) root and rhizome*	KA-induced *in vivo* excitotoxicity and primary cortical cultures	[136]
	Naringin (flavonoid)	Grapefruit and citrus fruit	KA-induced *in vivo* excitotoxicity	[22]
	Baicalin	*Scutellaria baicalensis*	KA-induced *in vivo* excitotoxicity	[137]
	Blueberry polyphenols	Blueberry	KA-induced *in vivo *and *in vitro *FaO rat hepatoma cells excitotoxicity	[94, 138]

Anticonvulsion and antiepileptic	Chongmyungtang	*Acorus gramineus, Polygala tenuifolia*, and* Poria cocos*	KA-induced *in vivo* excitotoxicity	[113]
	Decursin (purified from ethanol extract of the *Angelica gigas * Nakai root)	*Angelica gigas * Nakai	KA-induced *in vivo* and *in vitro* primary hippocampal neuronal cells excitotoxicity	[108]
	Acetyl-11-keto-*β*-boswellic acid (AKBA)	*Boswellia serrata*	KA-induced *in vivo* excitotoxicity	[95]
	Green tea leaf extract and gallic acid	*Camellia sinensis*	KA-induced *in vivo* excitotoxicity and KA-induced *in vitro *PC12 cells excitotoxicity	[74]
	Pu-Erh tea leaf extract and gamma-aminobutyric acid (GABA)	*Camellia sinensis *var. *assamica*	KA-induced *in vivo* excitotoxicity and KA-induced *in vitro *PC12 cells excitotoxicity	[75]
	Capsaicin	*Capsicum* (hot pepper)	KA-induced *in vivo* excitotoxicity	[121]
	Sinapic acid	Brassicaceae	KA-induced *in vivo* excitotoxicity	[139]
	Apigenin (flavone)	*Cirsium japonicum *and* Carduus crispus*	KA-induced *in vivo* and *in vitro* primary hippocampal neuronal cells excitotoxicity	[122]
	Ethanolic extract of *Desmodium adscendens*	*Desmodium adscendens*	KA-induced *in vivo* excitotoxicity	[140]
	*Gastrodia elata* extract	*Gastrodia elata*	KA-induced *in vivo* excitotoxicity	[70, 125]
	Naringin (flavonoid)	Grapefruit and citrus fruit	KA-induced *in vivo* excitotoxicity	[22]
	Vineatrol	Grapes	KA-induced *in vivo* excitotoxicity	[126]
	Ginsenosides	*Panax ginseng *(Asian)	KA-induced *in vivo* excitotoxicity	[65]
	Rb ginsenosides (Rb extract)	*Panax quinquefolius (*America)	KA-induced *in vivo* excitotoxicity	[26]
	Butanol fraction of methanol extract from *Petasite japonicus *Max. leaves (BMP) and its subfractions (BMP-I or BMP-II)	*Petasite japonicus *(Sieb. et Zucc.) Maxim. (Compositae) leaves	KA-induced *in vivo* excitotoxicity	[128]
	Petaslignolide A (lignin glycoside) butanol fraction of methanol extract from *Petasite japonicus* (Sieb. et Zucc.) Maxim. (Compositae) leaves	*Petasite japonicus* (Sieb. et Zucc.) Maxim. (Compositae) leaves	KA-induced *in vivo* excitotoxicity	[129]
	Rhynchophylline and *Uncaria rhynchophylla *extract	*Uncaria rhynchophylla*	KA-induced *in vivo* and *in vitro *excitotoxicity	[29, 70]
	*Gastrodia elata* extract	*Gastrodia elata*	KA-induced *in vivo* excitotoxicity	[70, 125]
	trans-Resveratrol	Red Grapes	KA-induced *in vivo* excitotoxicity	[130, 131]
	Branch and leaf ethanol extracts of *Vitis thunbergii* var. *taiwaniana*	*Vitis thunbergii *var. *taiwaniana*	KA-induced *in vivo* and *in vitro* BV2 cells excitotoxicity	[133]

Modulation of apoptosis-regulatory genes/proteins and signaling pathways	Curcumin	*Curcuma longa* Linn (turmeric)	KA-induced *in vivo* excitotoxicity	[110, 123]
	Anthocyanins	*Glycine max *(L.) Merr. seed (Korean black bean)	KA-induced *in vitro * HT22 cells and primary hippocampal neuronal cells excitotoxicity	[111]
	Ethanolic-extracted propolis	Honey bee propolis	KA-induced *in vivo* excitotoxicity	[84]
	Baicalin	*Scutellaria baicalensis*	KA-induced *in vivo* excitotoxicity	[137]
	Green tea leaf extract and gallic acid	*Camellia sinensis*	KA-induced *in vivo* excitotoxicity and KA-induced *in vitro *PC12 cells excitotoxicity	[74]
	Pu-Erh tea leaf extract and gamma-aminobutyric acid (GABA)	*Camellia sinensis *var. *assamica*	KA-induced *in vivo* excitotoxicity and KA-induced *in vitro *PC12 cells excitotoxicity	[75]
	*Gastrodia elata* extract	*Gastrodia elata*	KA-induced *in vivo* excitotoxicity	[70, 125]
	Rhynchophylline and *Uncaria rhynchophylla *extract	*Uncaria rhynchophylla*	KA-induced *in vivo* and *in vitro *excitotoxicity	[104]

Cognitive enhancer	Blueberry polyphenols	Blueberry	KA-induced *in vivo *and *in vitro *FaO rat hepatoma cells excitotoxicity	[94, 138]
	Sinapic acid	Brassicaceae	KA-induced *in vivo* excitotoxicity	[139]
	Galantamine	*Galanthus nivalis*	KA-induced *in vivo* excitotoxicity	[20]
	*Gastrodia elata* extract	*Gastrodia elata*	KA-induced *in vivo* excitotoxicity	[70, 125]
	Licorice flavonoids extracted→liquiritigenin, isoliquiritigenin, and liquiritin	*Glycyrrhiza uralensis*	KA-induced *in vivo* excitotoxicity	[18]
	Naringin (flavonoid)	Grapefruit and citrus fruit	KA-induced *in vivo* excitotoxicity	[22]

Manipulation of pro- and anti-inflammatory cytokines	Blueberry polyphenols	Blueberry	KA-induced *in vivo *and *in vitro *FaO rat hepatoma cells excitotoxicity	[94, 138]

Manipulation of glial activation and inflammatory cytokines	Chongmyungtang	*Acorus gramineus, Polygala tenuifolia*, and* Poria cocos*	KA-induced *in vivo* excitotoxicity	[113]
	Decursin (purified from ethanol extract of the *Angelica gigas * Nakai root)	*Angelica gigas * Nakai	KA-induced *in vivo* and *in vitro* primary hippocampal neuronal cells excitotoxicity	[108]
	Sinapic acid	Brassicaceae	KA-induced *in vivo* excitotoxicity	[139]
	Acacetin (flavone)	*Clerodendrum inerme* (L.) Gaertn (Ci)	KA-induced *in vivo* excitotoxicity	[141]
	Curcumin	*Curcuma longa* Linn (turmeric)	KA-induced *in vivo* excitotoxicity	[110, 123]
	RVH-1 (stigma-4-en-3-one) and RVH-2 (stigma-4-en-3,6-dione)	Detoxified *Rhus verniciflua*	KA-induced *in vivo* excitotoxicity	[142]
	Glycyrrhizin (triterpene)	*Glycyrrhiza glabra (licorice) root and rhizome*	KA-induced *in vivo* excitotoxicity and primary cortical cultures	[136]
	trans-Resveratrol	Red Grapes	KA-induced *in vivo* excitotoxicity	[130, 131]
	Rhynchophylline and *Uncaria rhynchophylla *extract	*Uncaria rhynchophylla*	KA-induced *in vivo* and *in vitro *excitotoxicity	[29, 72]

Enhancing mitochondrial functions	Ursolic acid	*Cynomorium songaricum *Rupr. (Souyang)	KA-induced *in vitro *hippocampal neuronexcitotoxicity	[124]
	Galantamine	*Galanthus nivalis*	KA-induced *in vivo* excitotoxicity	[20]
	Ginsenosides	*Panax ginseng *(Asian)	KA-induced *in vivo* excitotoxicity	[65]

Inhibition of NO by NOS inhibitors	Branch and leaf ethanol extracts of *Vitis thunbergii* var. *taiwaniana*	*Vitis thunbergii *var. *taiwaniana*	KA-induced *in vivo* and *in vitro* BV2 cells excitotoxicity	[133]

Combating excitotoxicity	*Ginkgo biloba* leaf extract	*Ginkgo biloba*	KA-induced *in vitro* cerebellar neuronal cells excitotoxicity	[116]
